# Neurophysiological Examination for the Diagnosis of Orofacial Pain and Temporomandibular Disorders: A Literature Review

**DOI:** 10.3390/diagnostics15081035

**Published:** 2025-04-18

**Authors:** Loredana Raciti, Martina Ferrillo, Antonio Ammendolia, Gianfranco Raciti, Claudio Curci, Dario Calafiore, Maria Pia Onesta, Rocco Salvatore Calabrò, Umile Giuseppe Longo, Alessandro de Sire

**Affiliations:** 1 Unità Spinale Unipolare, Azienda Ospedaliera per le Emergenze Cannizzaro, 98102 Catania, Italy; loredana.raciti79@gmail.com (L.R.); mp.onesta@gmail.com (M.P.O.); 2 Dentistry Unit, Department of Health Sciences, University of Catanzaro “Magna Graecia”, 88100 Catanzaro, Italy; martina.ferrillo@unicz.it; 3 Physical Medicine and Rehabilitation, Department of Medical and Surgical Sciences, University of Catanzaro “Magna Graecia”, 88100 Catanzaro, Italy; ammendolia@unicz.it (A.A.); gianfranco.raciti@gmail.com (G.R.); claudio.curci@asst-mantova.it (C.C.); 4 Research Center on Musculoskeletal Health, MusculoSkeletalHealth@UMG, University of Catanzaro “Magna Graecia”, 88100 Catanzaro, Italy; 5 Physical Medicine and Rehabilitation Unit, Department of Neurosciences, ASST Carlo Poma, 46100 Mantova, Italy; dario.calafiore@asst-mantova.it; 6 IRCCS Centro Neurolesi Bonino-Pulejo, 98124 Messina, Italy; roccos.calabro@irccsme.it; 7 Fondazione Policlinico Universitario Campus Bio-Medico, Via Alvaro del Portillo 200, 00128 Roma, Italy; g.longo@unicampus.it; 8 Research Unit of Orthopaedic and Trauma Surgery, Department of Medicine and Surgery, Università Campus Bio-Medico di Roma, Via Alvaro del Portillo 21, 00128 Roma, Italy

**Keywords:** temporomandibular disorders, neurophysiology, trigeminal nociceptive system, surface electromyography

## Abstract

Temporomandibular disorders (TMD) are a cluster of musculoskeletal conditions that involve the overall structures of jaw movements, including the temporomandibular joint, the masticatory muscles, and the surrounding structures. The etiology of TMD-related pain may be either central or peripheral, and differential diagnoses with other orofacial conditions are commonly required. Central pain etiology is associated with altered brain function linked to sensitization of pain-producing centers, whereas peripheral etiology of TMD is considered multifactorial, with some predisposing factors. Differentiating between neurological conditions and TMD requires a comprehensive clinical evaluation, as overlapping symptoms can complicate the diagnostic process. The aim of this review was to summarize the current literature about the role of neurophysiological examination in the management of orofacial pain and temporomandibular disorders to provide clear data that could be useful for clinical practice and for future clinical studies in this field.

## 1. Introduction

The orofacial region mediates exquisite sensitivity and an ability for sensory discrimination and presents several distinct and vital functions. Accordingly, the rich peripheral innervation and the large cortical representation areas for the sensory and motor orofacial functions reflect the importance of this region [1]. In this context, the orofacial region is prone to several acute and pain conditions, considering that the prevalence of chronic orofacial pain is in the 8% to 15% range [2].

One of the main conditions of orofacial pain is due to temporomandibular disorders (TMDs), a cluster of musculoskeletal conditions that involve the overall structures of jaw movements, including the temporomandibular joint (TMJ), the masticatory muscles, and the surrounding structures [3].

Prevalence of TMD is highest among individuals aged 18 to 60 years [4,5], with a greater susceptibility observed in women. This difference may be partially explained by hormonal fluctuations—particularly involving estrogen—which have been associated with pain modulation, especially in the temporomandibular joint and the orofacial region, rather than being identified as a direct cause of TMD [6]. Recent literature indicates that the current evidence is not sufficient to establish a definitive link between estrogen and the development of TMD [7]. Additional contributing factors may include a higher incidence of humoral and psychosocial disorders in women, as well as demographic data showing that, across all continents, the female population affected by TMD was on average 9% to 56% larger than the male population. The highest female-to-male ratio was reported in South America (1.56), and the lowest in Europe (1.09) [5]. TMDs encompass a group of musculoskeletal conditions involving the temporomandibular joint (TMJ), masticatory muscles, and associated structures. Pain is typically localized to the jaw, preauricular area, or temporal regions and is often associated with functional limitations such as reduced mandibular movement or TMJ sounds. Differential diagnosis is challenging due to overlapping symptoms with primary headache disorders, neuropathic conditions, and dystonias. Recent advances have shifted attention from purely mechanical etiologies toward neurophysiological mechanisms underlying TMD-related pain (TMD-P) [8,9,10,11].

The etiology of TMD-related pain (TMD-P) may be either central or peripheral [12]. Central pain etiology is associated with altered brain function linked to sensitization of pain-producing centers, marked by increased activation of the somatosensory cortex, anterior cingulate, and prefrontal cortex, and reduced thalamic activation in TMD patients [13,14]. In contrast, peripheral etiology of TMD is considered multifactorial, often involving prolonged use of the masticatory muscles such as the masseters (MMs) and anterior temporalis (ATs), grinding and clenching, repetitive trauma to the TMJ, and psychological disorders (e.g., anxiety and depressive syndromes) [13]. Peripheral sources of TMD pain emphasize the mechanical and functional aspects that contribute to muscle strain and joint dysfunction [15].

TMD are diagnosed primarily through patient history and clinical examination, sometimes supported by imaging such as ultrasound, which allows for dynamic, non-invasive assessment of joint function [16,17,18]. However, symptom overlap with neurological conditions—like trigeminal neuralgia, dystonia, or multiple sclerosis—often complicates diagnosis, requiring careful clinical evaluation and, when needed, additional tools like MRI or EMG to distinguish TMD from other orofacial pain sources [19,20,21,22,23,24,25].

Chronic TMD can cause structural and functional neuroplastic changes in the trigeminal system, resulting in central sensitization [14]. This leads to heightened responsiveness to stimuli, causing allodynia (pain from non-painful stimuli) or hyperalgesia (exaggerated pain response).

The neurophysiological basis of TMD involves a multifaceted interplay between peripheral and central nervous system (CNS) mechanisms, contributing to both acute and chronic pain manifestations [26]. To enhance differential diagnosis between CNS diseases and peripheral sources of TMD, electronic instruments like EMG muscle activity recordings are utilized. However, the somatosensory sensitivity in TMD patients has shown variable results, including hypersensitivity, hyposensitivity, or no change, neurophysiological studies of TMD remain limited, and debates continue regarding the criteria and understanding of TMD pain pathophysiology [27,28,29,30].

Indeed, the trigeminal nucleus caudalis, a critical relay station in the brainstem, integrates sensory information related to TMD. It receives inputs from the TMJ and masticatory muscles and from cervical regions, which may lead to referred pain. This convergence of sensory inputs contributes to the complexity of TMD pain, where TMJ-originating pain can be perceived in other regions, such as the head, neck, and shoulders. Such convergence also plays a role in central sensitization, where repeated pathway stimulation induces long-term changes in neuronal excitability and synaptic plasticity, sustaining pain even in the absence of peripheral stimuli [31].

Furthermore, TMD patients often exhibit a dysregulated autonomic nervous system, with increased sympathetic activity that may exacerbate muscle tension and joint dysfunction [32]. Psychological factors, including anxiety and depression, also influence pain perception, creating a bidirectional relationship between emotional well-being and TMD symptom severity. This underscores the importance of a biopsychosocial approach to treatment.

However, to date, there is still a lack of evidence that summarizes the role of neurophysiological examination in the differential diagnosis and management of orofacial pain and TMD.

Therefore, the aim of this review was to report the current literature about the role of neurophysiological examination in the management of orofacial pain and temporomandibular disorders to provide clear data that could be useful for clinical practice and for future clinical studies in this field.

## 2. Neurophysiology of the Trigeminal Pain Pathway

The trigeminal pain pathway is characterized by ascending and descending pathways [33]. Many TMD patients report increased sensitivity not just in the jaw area but in other regions served by the trigeminal nerve [34]. This suggests that TMD may influence broader trigeminal pathway sensitivity [1]. Noxious stimuli or overwhelming irritation of the orofacial region repetitively stimulate the peripheral nociceptors of the same area, and exacerbated pain signals are developed by the first-order neurons in the trigeminal nerve or cranial nerve number V (cnV) and transmitted to the trigeminal ganglia [33,35].

Then, pain signals are transmitted to the brainstem by the second-order neurons contained within the trigeminal nucleus caudalis. Therefore, anatomically, three groups of trigeminal nuclei inside the brainstem have been described [36,37]. The pars oralis and interpolars of the first spinal nucleus deliver tactile sensation in the orofacial area. The third spinal nucleus of the cnV is the pars caudalis or trigeminal nucleus caudalis, which carries pain perception of this involved area [38,39]. Lastly, the pain signals will be projected to the third-order neurons of the thalamus and cortex through the ventral trigeminothalamic tract [38,39,40].

The pain modulation process involves the descending pathway from the somatosensory cortex to the trigeminal nucleus caudalis [36]. Another way to modulate the pain pathway is the projection of the brainstem neurons of somatosensory signals through the periaqueductal gray, locus coeruleus, and rostral ventromedial medulla via a direct or indirect pathway [38]. The indirect pathway involves the central midbrain neurons corresponding to the periaqueductal gray, which modulate pain pathways indirectly via others brainstem nuclei, including the locus coeruleus, composed of noradrenergic neurons projecting to the trigeminal nucleus caudalis, and the rostral ventromedial medulla, a large region of the medulla that contains serotoninergic neurons [39]. Therefore, these neurotransmitters or enkephalin or opioid peptides are produced, leading to pain reduction [40]. A decrease in neurons on both sides of the brainstem, especially in the rostral ventromedial area, have been described as the origin of patients’ temporomandibular joint pain [2,41]. Recently, the pathophysiology of orofacial pain has been related to the impairment of the trigeminal pain pathway and modulation, modifying pain perception in the somatosensory cortex and associated somatosensory cortex, anterior cingulate gyrus, and prefrontal cortex [42,43].

## 3. Neurophysiology of TMD Pain

The neuropathophysiology underlying TMD with chronic pain has been better studied via fMRI research, analyzing structural and functional alterations in key brain networks and pathways [3,12,26,42,43,44]. The authors demonstrate that TMD-related pain involves widespread alterations in brain structure and function across sensory, emotional, and motor systems.

Findings showed changes in the trigemino-thalamo-cortical pathway, including the trigeminal nerve root, spinal trigeminal nucleus, thalamus, and primary somatosensory cortex (S1), which are involved in pain processing. Details on MRI findings in the recent literature are summarized in Table 1 [4,45].

Orofacial pain is a common symptom with several multifaceted origins. Thus, differential diagnostic procedures are often needed to be able to identify alternative painful orofacial conditions [43]. In 2020, a new International Classification of Orofacial Pain (ICOP) was published based on the hierarchical classification model of the International Classification of Headache Disorders (see Table 2) [44,45].

While anatomical classifications such as the ICOP [44,45] offer a structured framework for diagnosis—categorizing pain based on involved structures, symptom duration (acute vs. chronic), and underlying mechanisms—they should be interpreted in the context of evolving insights into pain neurobiology. Acute OFP may involve predominantly peripheral nociceptive processes, whereas chronic pain often implicates central sensitization and neuroplastic changes that sustain the pain experience over time (see Table 3).

The etiology of TMDs is still debated, as studies have refuted the hypotheses suggesting muscle hyperactivity or central nervous system (CNS) overactivity in TMD patients [20,27]. Instead, the trigeminal nerve’s sensory nucleus, particularly the spinal trigeminal nucleus in the brainstem, is crucial for processing pain and sensory information in TMD. Patients with TMD often exhibit increased excitability or central sensitization within this nucleus, which contribute to persistent symptoms even in the absence of ongoing peripheral damage. This sensitization disrupts motor control in the trigeminal nerve, specifically affecting the masseter muscle, which plays a key role in chewing. As a result, it can lead to muscle stiffness or spasms, further exacerbating the pain cycle [12,20,27]. This phenomenon amplifies pain perception and contributes to persistent symptoms, even in the absence of ongoing peripheral damage.

Neurophysiological studies have used tools like trigeminal laser-evoked potentials (LEPs) and laser silent periods (LSP) to explore nociceptive pathways, finding suppressed cortical responses and reflex hypo-excitability in TMD patients. These findings indicate dysfunction in trigeminal nociceptive processing [46]. Electrophysiological tests, especially the blink reflex (BR), have proven essential for differentiating CNS conditions from peripheral TMD sources. The blink reflex (BR) test evaluates the trigeminal and facial nerves and can reveal abnormalities, such as delayed or absent R2 components, in TMD patients. Such changes reflect central sensitization, a hallmark of chronic pain, and are linked to increased neuronal excitability in brainstem pathways (see Table 4).

Studies have also shown that psychological stress and anxiety correlate with BR abnormalities, supporting the biopsychosocial model of TMD [46,47,48,49]. The biopsychosocial model of TMD, which considers the complex interactions of biological mechanisms, psychological states (such as stress, anxiety, or depression), and social influences, provides a comprehensive framework for understanding and managing chronic orofacial pain conditions [48].

Psychological stress and anxiety have been shown to influence blink reflex outcomes, suggesting that emotional factors contribute to altered pain perception and central processing [48,49]. This integration of psychological, neurological, and physiological dimensions allows for a more comprehensive approach to understanding chronic OFP.

Functional imaging, such as fMRI and PET, highlights hyperactivity in brainstem regions processing trigeminal pain. This hyperactivity aligns with findings of altered somatosensory-evoked potentials (SSEPs) and disrupted sensory signal transmission in TMD patients [45,50]. Additionally, jaw reflex analyses reveal variable inhibition phases, with silent periods (SPs) showing prolonged or unchanged durations in TMD patients compared to controls [51].

In this context, clinical physiatric and gnathologic evaluations may also be useful in the characterization of patients with chronic TMD in terms of existing hyperalgesia to cold, heat, and pressure stimuli. Indeed, a recent study [52] showed that clinical assessments using tools like the PainDETECT questionnaire, along with physiatric and gnathologic evaluations, are useful for identifying signs of hyperalgesia to thermal and pressure stimuli, further distinguishing between acute and chronic pain states and contributing to a personalized diagnostic profile.

## 4. Surface Electromyography for Temporomandibular Disorders

The recognition of muscle disfunction is essential in patients affected by TMD, especially to guide physicians in performing correct decision-making processes for rehabilitation. Surface EMG (sEMG) is a non-invasive, accessible, and reliable tool to evaluate muscle function by analyzing the electrical signals produced during muscle contractions. sEMG might provide valuable clinical data on the activity of certain muscles at rest and under activation [53,54] to identify muscle hyper- or hypoactivation. However, the diagnostic efficacy of sEMG in assessing individuals with painful TMD remains still unclear [55,56].

Studies exploring the utility of sEMG in detecting muscle abnormalities in TMD patients and assessing treatment efficacy have suggested that factors such as electrode placement, impedance imbalances, and device limitations might reduce predictive validity [57]. Over the past decades, sEMG has benefited from substantial methodological refinement, especially regarding electrode placement, signal filtering, and standardization protocols. Since the 1960s, authors [58,59,60,61,62,63,64,65,66,67] have laid the groundwork for reliable sEMG procedures in dentistry. These efforts were further consolidated with the publication of the SENIAM guidelines [60], which provide clear recommendations for electrode type, positioning, and interelectrode distance. More recently, specific sEMG guidelines tailored to dental applications were presented by Zieliński and Gawda [67], emphasizing both technical standards and clinical applicability.

While sEMG remains a valuable tool for assessing muscle function, some limitations may still arise in signal interpretation, patient-specific variability, or equipment sensitivity. However, these challenges should not be attributed to the lack of standardized placement procedures, which are now well-established and implemented in dental research.

Recent literature has emphasized the need for greater methodological consistency in the use of sEMG in the assessment of TMD [59,60,61,62]. The SENIAM project (Surface Electromyography for the Non-Invasive Assessment of Muscles) established technical recommendations aimed at improving the reliability, reproducibility, and comparability of sEMG recordings across research and clinical settings [59,60,61,62]. The SENIAM guidelines provide standardized instructions regarding electrode type, placement, interelectrode distance, and signal filtering. Specifically, they recommend positioning bipolar surface electrodes along the muscle fiber direction—avoiding tendon insertions—with an interelectrode distance of 20 mm and using bandpass filters typically between 20 and 500 Hz. Electrode placement should follow the SENIAM recommendations, particularly over the masseter and temporalis muscles. In addition, standardized testing conditions are advised, such as a seated patient posture, 10-s rest recordings, and Maximum Voluntary Contraction (MVC) protocols consisting of, for example, two contractions of 3 s with 3 s pauses, or three contractions of 5 s with 60 s intervals. These principles have become widely adopted as a methodological foundation for sEMG studies involving masticatory muscles [62].

A review by Zieliński and Gawda [67] builds upon this framework, presenting an in-depth overview of the historical development, clinical relevance, and future potential of sEMG in dentistry and proposing technical parameters tailored to the assessment of TMDs. In particular, the authors proposed a set of technical parameters aimed at standardizing the collection and analysis of electromyographic signals. These recommendations, summarized in Table 5, include specifications for sampling rate, signal bandwidth, noise thresholds, electrode placement, and patient positioning, as well as measurement protocols such as resting and maximum voluntary contraction (MVC) trials. The goal was to enhance the diagnostic precision of sEMG and promote comparability across studies.

While these recommendations reflect current best practices, they are not yet globally standardized and should be considered evolving guidelines rather than definitive diagnostic criteria. The authors emphasize the need for further high-quality clinical trials to validate these protocols and clarify sEMG’s role in TMD diagnosis. Additionally, a deeper understanding of trigeminal pathway changes in TMD can support better diagnostic outcomes.

A systematic review and meta-analysis by Dinsdale et al. showed that masseter and temporalis activity was significantly higher at rest (*p*-value = 0.05 and *p*-value = < 0.0001, respectively) but lower during brief maximal clenching (*p*-value = 0.005 and *p*-value = < 0.04, respectively) in TMD patients compared to healthy controls, thus reflecting sensory–motor imbalances and pain-induced movement alterations [54].

Prolonged silent periods in the masseter and temporalis muscles during clenching and tapping further highlight TMD-induced motor dysfunctions, which are often alleviated by occlusal therapy [5,68]. Research has also demonstrated the role of sEMG in tracking treatment outcomes. However, limitations such as signal interference and device constraints reduce its predictive validity for TMD diagnosis [68]. Furthermore, inconsistent clinical criteria and the lack of standardization in data interpretation complicate its broader diagnostic application.

Recent studies have expanded its use alongside other neurophysiological tools, such as trigeminal reflex testing, to better differentiate TMD subtypes like muscle-related disorders, disc displacement, and dystonic conditions [57,68]. Recognizing these distinctions is essential for tailoring rehabilitation strategies and advancing objective, quantitative evaluations of TMD pathophysiology. However, it should be noted that in the last year, there was not a clear definition of the role of neurophysiological examination (i.e., sEMG) in the diagnosis of TMD.

The treatment of TMD involves both peripheral and central mechanisms that sustain pain and dysfunction. Effective management targets not only the source of pain but also neurophysiological changes contributing to chronic symptoms (see Table 6). Treatment approaches could be synthesized as follows:

- Pharmacological: NSAIDs and muscle relaxants reduce inflammation and hyperactivity, while tricyclics and SNRIs modulate descending pain pathways, reducing hyperalgesia and central sensitization.
- Physical therapy and behavioral interventions: Jaw exercises and postural correction relieve muscle tension. Cognitive–Behavioral Therapy (CBT) helps manage stress-related pain, with hypnosis showing a 47% pain reduction in TMD patients but no significant BR changes.
- Occlusal splints and bruxism monitoring: Splints reduce muscle hyperactivity and nociceptive input. The Bruxoff device, combining EMG and ECG, improves bruxism detection, correlating well with polysomnography.

Teledentistry has emerged as a valuable tool, offering remote monitoring and rehabilitation, particularly useful during the COVID-19 pandemic.

Ultimately, an interdisciplinary approach combining physical, psychological, and neurophysiological interventions is essential for optimizing pain relief and improving TMJ function and patient outcomes.

## 5. Future Directions

The presence of serious neurological or systemic pathologies can complicate differential diagnosis of TMD, particularly in early stages. Over the past few decades, sEMG has undergone notable methodological advancements, particularly regarding electrode placement, signal processing, and standardized testing protocols. Seminal contributions by Ferrario and Sforza have laid the groundwork for reliable sEMG procedures in dentistry, especially in the analysis of masticatory muscle activity in both physiological and pathological conditions [65]. These developments were further consolidated through the SENIAM project (Surface Electromyography for the Non-Invasive Assessment of Muscles), which provided a comprehensive set of technical recommendations aimed at improving the reproducibility, reliability, and comparability of sEMG data across clinical and research contexts [66,67,69].

The SENIAM guidelines, widely referenced in the literature, include recommendations such as positioning bipolar surface electrodes along the muscle fiber direction—avoiding tendon insertions—maintaining a 20 mm interelectrode distance, and applying bandpass filters typically between 20 and 500 Hz. In addition, they advise standardized testing conditions, such as seated patient posture, rest recordings, and Maximum Voluntary Contraction (MVC) protocols. These principles have become foundational in many studies involving masticatory muscles, particularly the masseter and temporalis.

Building upon this framework, Zieliński and Gawda [67] proposed a set of sEMG parameters specifically tailored to dental applications and TMD assessment. These proposals, summarized from the literature and clinical experience, represent current best practices aimed at improving the methodological rigor of electromyographic research in dentistry.

However, these parameters should not be interpreted as fixed diagnostic criteria. As emphasized by the authors themselves, the recommendations remain expert-driven proposals rather than internationally recognized standards. While certain aspects—such as the use of high sampling rates and SENIAM-based electrode positioning—are frequently applied in research settings, a universal consensus regarding their diagnostic application in TMD is still lacking.

Moreover, the presence of serious neurological or systemic pathologies can further complicate the differential diagnosis of TMD, particularly in its early stages. This highlights the need for caution when interpreting sEMG data, especially in clinically ambiguous or complex cases. The potential for overlap in symptomatology between TMD and other orofacial or neuropathic pain conditions underscores the necessity of integrating sEMG findings with comprehensive clinical evaluation.

In this context, while the technical recommendations proposed by Zieliński and Gawda [67] contribute significantly to standardizing sEMG-based studies, they should be applied with discretion and adapted to the specific clinical or research scenario. Further high-quality clinical trials are required to validate these protocols across diverse patient populations and TMD subtypes and to clarify the diagnostic and prognostic value of sEMG under standardized conditions.

Understanding changes in the trigeminal pathway in TMD can improve diagnostic accuracy. Assessing the trigeminal blink reflex, sEMG, SSEPs can give clinicians insight into the extent of central sensitization and neural changes, although the scientific literature currently lacks strong studies in this field.

The neurophysiology of TMD involves complex interactions between peripheral nociceptive mechanisms, central sensitization, and psychological factors, also considering the different therapies for TMD (e.g., exercise, physical agent modalities, occlusal splints, telerehabilitation, etc.) [66,67,69,70,71,72,73,74,75]. Future advances in neuroimaging and neurochemical analysis are continuing to shed light on these complex pathways, improving the management of orofacial pain through more targeted and effective interventions. An interdisciplinary approach involving neurologists, oral and maxillofacial specialists, orofacial pain specialists, physical therapists, and pain management professionals is often essential for accurate diagnosis and effective treatment planning.

## 6. Conclusions

The present review aimed to investigate the scientific literature on the complex management of orofacial pain, starting from the knowledge of neurophysiology of these pathways. The initial assessment should begin with a thorough patient history, emphasizing the onset, triggering factors, and evolution of symptoms, which is essential for guiding the diagnostic process. Physical examination should include palpation of the temporomandibular joint and associated muscles, assessment of jaw range of motion, and evaluation of cranial nerve function. Targeted imaging, such as MRI or CT scans, should also be employed. Moreover, ultrasound enables dynamic assessment, allowing clinicians to observe joint movement during functional activities. This dynamic capability provides valuable insights into TMJ disorders, enhancing diagnostic precision and contributing to a more comprehensive understanding of the condition. Neurophysiological testing, including EMG or nerve conduction studies, can further refine the differential diagnosis. It is well known that chronic TMD could be considered as a condition of chronic primary pain related to dysfunction of the CNS, likely through the central sensitization.

Chronic activation of this pathway can result in central sensitization, characterized by increased neuronal responsiveness and amplified pain signals. This phenomenon is often associated with widespread pain, sleep disturbances, and an elevated stress response, all of which contribute to the chronic nature of TMD. Thus, central sensitization in TMD involves synaptic strength changes and altered activity in pain-processing neurons. Activated microglia and astrocytes release pro-inflammatory cytokines and other mediators that increase neuronal excitability, thus maintaining central sensitization.

These imaging techniques reveal altered activation patterns in brain regions involved in pain modulation, such as the anterior cingulate cortex, insula, and prefrontal cortex, highlighting central nervous system changes in TMD. Altered connectivity between these regions has also been observed, suggesting that the network dynamics of pain perception and emotional regulation are disrupted in TMD patients, potentially intensifying both sensory and affective dimensions of pain. In conclusion, the neurophysiological basis of TMD reflects an intricate interplay between peripheral and central nervous system mechanisms, which contribute to both acute and chronic pain. A deeper understanding of this neurophysiology is essential to advance effective treatments.

## Figures and Tables

**Table 1:** Summary of magnetic resonance imaging findings about neurophysiological basis of temporomandibular disorders.

Authors	Aspect	Findings
Yin et al., 2020 [12] Mills et al., 2021 [26]	Trigemino-thalamo-cortical pathway	Alterations in the trigeminal nerve root, spinal trigeminal nucleus, thalamus, and primary somatosensory cortex (S1). Structural changes include reduced/increased gray matter volume and disrupted white matter microstructure.
Yin et al., 2020 [12] Domin et al., 2021 [42]	Lateral and medial pain systems	Changes in anterior insula and anterior cingulate cortex (ACC) connectivity and volume, correlating with pain intensity and duration. Highlighted GMV reductions in ACC and medial prefrontal cortex (mPFC).
Yin et al., 2020 [12]	Default Mode Network (DMN)	Increased functional connectivity linked to heightened pain rumination and emotional responses.
Yin et al., 2020 [12] Mills et al., 2021 [26]	Descending pain modulation systems	Structural alterations in the periaqueductal gray–raphe magnus pathway impair descending pain inhibition. Functional connectivity changes in the rostral ventromedial medulla (RVM), subnucleus reticularis dorsalis, and spinal trigeminal nucleus suggest enhanced pain signal facilitation.
Yin et al., 2020 [12]	Motor system	Abnormal activations in primary motor cortex and supplementary motor areas lead to disrupted motor control and jaw dysfunction.
Yin et al., 2020 [12]	Neurochemical imbalances	Altered glutamate and choline levels in the insula reflect disrupted pain processing.
Yin et al., 2020 [12] Lam et al., 2024 [43]	Abnormal brain responses	Increased activity in anterior cingulate cortex and amygdala in response to mechanical stimuli. Enhanced thalamic involvement correlates with pain intensity and disability.
Yin et al., 2020 [12]	Effects of splint therapy	Functional reorganization observed with improved symptoms and normalized brain activity in motor and pain-processing regions.

**Table 2:** International Classification of Orofacial Pain (ICOP) 2020—main categories of orofacial pain.

Category	Description
1. Dentoalveolar and related structures	Pain arising from teeth, periodontium, or surrounding tissues
2. Myofascial orofacial pain	Pain from masticatory muscles, often associated with TMD
3. Temporomandibular joint (TMJ) pain	Pain originating from the TMJ and its components
4. Neuropathic orofacial pain	Pain caused by lesion or disease of the somatosensory nervous system
5. Orofacial pain resembling primary headache disorders	Pain mimicking migraine, cluster, or tension-type headache
6. Idiopathic orofacial pain	Persistent pain without clear structural or neurological cause
Psychosocial assessment	Evaluation of psychological and social factors influencing pain perception

Legend: This table outlines the six major diagnostic categories established in the hierarchical classification model of the International Classification of Orofacial Pain (ICOP), which guides clinicians in identifying and differentiating the various types of orofacial pain. Moreover, the other three subgroups include orofacial pains resembling presentations of primary headaches, idiopathic pain, and psychosocial assessment of patients with orofacial pain in the orofacial region. Thus, the ICOP is stratified by six main categories that are subdivided into subcategories, plus one section on the assessment of psychosocial factors relevant to orofacial pain [44,45].

**Table 3:** Orofacial pain: duration and pathophysiology.

Classification	Definition	Neurophysiological Mechanisms
Acute pain	Lasts less than 3 months	Mainly peripheral nociceptive activation
Chronic pain	Persists ≥ 3 months	Central sensitization, neuroplastic changes
Nociceptive pain	Related to tissue damage or inflammation	Activation of nociceptors (e.g., in TMJ, muscles)
Neuropathic pain	Caused by nerve injury or disease	Abnormal somatosensory processing, ectopic activity
Inflammatory pain	Due to immune response and tissue inflammation	Peripheral sensitization with possible central effects

Legend: This table summarizes the primary distinctions in orofacial pain based on duration (acute vs. chronic) and underlying pathophysiological mechanisms (nociceptive, inflammatory, neuropathic), with emphasis on their neurobiological correlates.

**Table 4:** Neurophysiological tools in TMD and their diagnostic significance.

Tool	What It Measures	Findings in TMD Patients	Interpretation
Laser-Evoked Potentials (LEPs)	Cortical response to nociceptive stimuli via Aδ and C fibers	Reduced cortical amplitudes and delayed latencies	Impaired cortical nociceptive processing
Laser Silent Period (LSP)	Inhibitory spinal reflex following laser stimulus	Shortened or absent silent period	Brainstem/spinal disinhibition, altered nociceptive reflexes
Blink Reflex (BR)	Brainstem reflex involving trigeminal and facial nerves (R1, R2)	Delayed or absent R2 component	Central sensitization and brainstem hyperexcitability
Somatosensory-Evoked Potentials (SSEPs)	Cortical response to peripheral somatosensory stimuli	Altered waveform morphology, prolonged latencies	Disrupted sensory transmission and central integration
Jaw Reflex and Silent Period (SP)	Reflex motor inhibition following mandibular stimulation	Prolonged or unchanged SP duration	Altered motor control, possible central modulation dysfunction

Legend: This table reviews key neurophysiological tools used to assess trigeminal and brainstem function in patients with temporomandibular disorders (TMDs), highlighting typical abnormalities and their implications for identifying central sensitization and altered pain processing.

**Table 5:** Recommended sEMG parameters for muscle assessment.

Parameter	Recommended Value/Description
Sampling rate	≥2000 Hz
Bandwidth	20–500 Hz (for masticatory muscles)
Baseline noise	<1 µV RMS
Input impedance	>100 MΩ
Common Mode Rejection Ratio (CMRR)	>100 dB (ideally > 130 dB at 60 Hz)
Electrode placement	According to SENIAM guidelines (e.g., over masseter and temporalis muscles)
Patient positioning	Seated posture, head in natural position
Resting recording duration	10 s
MVC protocol	2 × 3 s with 3 s pause, or 3 × 5 s with 60 s pause

**Table 6:** Neurophysiology changes after treatment of TMD.

Treatment Type	Mechanism	Effects	References
Pharmacological Interventions			
NSAIDs and muscle relaxants	NSAIDs reduce local inflammation and peripheral nociceptor activation, leading to reduced pain transmission to the CNS	Decreases peripheral sensitization, indirectly lowering central sensitization and hyperactivity in muscles	Szyszka-Sommerfeld et al., 2022 [59]
Antidepressants	Modulate descending inhibitory pathways, regulate neurotransmitters like serotonin and norepinephrine	Reduces central sensitization and hyperalgesia	de Sousa BM et al., 2024 [60]
Physical therapy			
Exercises and manual therapy	Improves jaw mobility and decreases muscle tension, as reflected in reduced muscle hyperactivity in sEMG	Alleviates TMD symptoms by targeting abnormal muscle activation	Dinsdale et al., 2021 [54]
Postural correction	Reduces abnormal loading on TMJ and masticatory muscles, decreasing nociceptive input from overloaded structures	Lowers both peripheral and central sensitization	Al-Saleh et al., 2012 [68]
Cognitive–Behavioral Therapy (CBT)			
Managing psychosocial factors	Addresses chronic pain-related psychosocial factors, reducing activity in stress-related brain regions	Lowers stress-related activity in amygdala, normalizes ACC and insula hyperactivity	Koh and Drummond, 2006 [49]
Biofeedback integration	Uses sEMG in CBT to help patients control muscle activity consciously	Improves muscle relaxation, decreases pain perception	Szyszka-Sommerfeld et al., 2023 [62]
Splint therapy			
Stabilization splints	Reduces excessive muscle activity in masseter and temporalis muscles	Lowers nociceptive input, decreases central sensitization	Suvinen et al., 2007 [57]
Reduction in bruxism	Reduces bruxism, thereby decreasing repetitive mechanical stress on TMJ and masticatory muscles	Alleviates pain and other TMD symptoms	Frisardi et al., 2012 [64]

Legend: Nonsteroidal anti-inflammatory drugs (NSAIDs); CNS: Central Nervous System; sEMG: surface electromyography; TMJ: temporomandibular joint; TMD: temporomandibular disorders; ACC: anterior cingulate cortex.

## Data Availability

The data that support the findings of this study are available from the corresponding author upon reasonable request.
